# Layered Double Hydroxides Precursor as Chloride Inhibitor: Synthesis, Characterization, Assessment of Chloride Adsorption Performance

**DOI:** 10.3390/ma11122537

**Published:** 2018-12-13

**Authors:** Lin Chi, Zheng Wang, Youfang Zhou, Shuang Lu, Yan Yao

**Affiliations:** 1 School of Civil Engineering, Harbin Institute of Technology, Harbin 150001, China; chilin8958@163.com (L.C.); wangz@163.com (Z.W.); 15692213712@163.com (Y.Z.); 2 Key Lab of Structures Dynamic Behavior and Control of the Ministry of Education, Harbin Institute of Technology, Harbin 150090, China; 3 Key Lab of Smart Prevention and Mitigation of Civil Engineering Disasters of the Ministry of Industry and Information Technology, Harbin Institute of Technology, Harbin 150090, China; 4 China Building Materials Academy, Beijing 100024, China; yy@cnbm.com.cn

**Keywords:** layered double hydroxides, chloride ion adsorption, corrosion inhibitor

## Abstract

In this study, the chloride adsorption behaviors of CaAl-Cl LDH precursors with various Ca:Al ratios were investigated. The optimal chloride ion removal rate was 87.06% due to the formation of hydrocalumite. The chloride adsorption products of CaAl-Cl LDH precursors were further characterized by X-ray diffraction analysis and atomic structure analysis, the adsorption mechanism was considered to be co-precipitate process. The chloride adsorption behaviors of cementitious materials blended with CaAl-Cl LDH precursors were further investigated. Leaching test according to Test Code for Hydraulic Concrete (SL352-2006) was performed to testify the stability of chloride ions in the mortar. The results show that more than 98.3% chloride ions were immobilized in cement mortar blended with CaAl-Cl LDH precursor and cannot be easily released again. The inhibition performance of steel in the electrolytes with/without CaAl LDH precursor was investigated by using electrochemical measurements. The results indicate that CaAl LDH precursor can effectively protect the passive film on steel surface by chloride adsorption. Considering the high anion exchange capacities of the LDHs, synthesized chloride adsorbent precursor can be applied as new inhibitors blended in cementitious materials to prevent the chloride-induced deterioration. Moreover, the application of chloride adsorption on CaAl-Cl LDH could also be of interest for the application of seawater blended concrete.

## 1. Introduction

Concrete structures can be easily deteriorated by chloride and thus failed to serve the predetermined service lives in marine environments. The corrosion occurs when sufficient amounts of chloride ions have been accumulated onto the surface of the embedded steel. Generally, the aggression of chloride ions into the concrete is time consuming for the well protected concrete cover. To enhance the concrete properties, several kinds of inhibitors have been developed and applied in the practical engineering projects [1]. Such kind of inhibitors are usually presented as a thin and weak inertia film that are just attached on the surface of the steel bars to prevent chloride attack, correspondingly serious local corrosion will occur on the unprotected area. Comparatively, layered double hydroxides (LDHs) are found to be effective chloride adsorbents, effectively preventing chloride-induced deterioration in reinforced cementitious materials [2,3].

The hydrocalumite-like structure monosulfaluminate phases (AFm) with a stoichiometric formula of [Ca_2_(Al, Fe)(OH)_6_)]·X·nH_2_O have the highest chloride-binding capacity among the mainly hydration products (e.g., portlandite, ettringite, and tobermorite) of Portland cement, which can be classified into the wider LDH family. As a result of the reaction with the existing AFm phases, chloride ions are adsorbed and thus stabilized in the Friedel’s salt. Nevertheless, the theoretical and practical amount of chloride by Cl-AFm phases are less than the synthetized LDH [4]. Since the experiment is not performed in a dilute solution, but a cement matrix, the effective binding capacity of AFm phases in the cement matrix becomes much less with the presence of other competitive anions in the pore solution. Nitrite type hydrocalumite is another synthetic LDH-like anion adsorbent that could absorb chloride ion effectively and release nitrite ions at the same time, which could be applied as a potential corrosion inhibitor [5,6]. Considering the high anion exchange capacities of the LDHs, Tatematsu and Sasaki [7] admixed LDHs phases in concrete as a chloride ions adsorbent. S. Yoon et al. showed that the use of calcined layered double hydroxides in cementitious materials can have extraordinary potential in preventing the chlorid-induced deterioration of the reinforced concrete [8].

Even though synthetized LDHs are effective absorbents that have a large chloride threshold level and can prevent chloride-induced deterioration in reinforced concrete, unfortunately it may slightly reduce the compressive strength [9]. Moreover, the amount of the synthesized LDH is quite limited for the large volume concrete in the construction project. Therefore, the ultra-high lime with aluminum process is a relative low cost and high effective method to synthesize CaAl LDH, by admixing lime and calcium aluminate in the chlorinated solution and precipitating with the chloride in the form of the calcium chloroaluminate (Ca_4_Al_2_Cl_2_(OH)_12_) [10,11]. In addition, the chemical constitution of CaAl LDH can be classified into Cl-AFm phases, which is more compatible with the cement-based materials. However, synthesized chloride adsorbent precursor served as a new inhibitor blended in cementitious materials to prevent the chloride-induced deterioration is rarely mentioned. The purpose of this study was to synthesize and use hydrocalumite-precursor for efficient chloride sorption in aqueous media. The chloride sorption capacity was achieved by the formation of hydrocalumite. Furthermore, the CaAl LDH precursor can be applied as a new-found chloride inhibitor blended in cementitious materials to prevent the chloride-induced deterioration or the application of seawater blended concrete.

## 2. Materials and Methods

### 2.1. Synthesis of CaAl-Cl LDH

All chemicals used in this study are of analytical grade and deionized water is used all through the experiments. CaAl-Cl LDH was synthesized by ultra high lime with aluminate process (UHLA) method as reported elsewhere [10]. Ca(OH)_2_ (Aladdin, Shanghai, China), NaAlO_2_ (Aladdin, Shanghai, China), and NaCl (Aladdin, Shanghai, China) ([Cl^−^] = 0.24 mol/L) with mole ratio n (Ca: Al: Cl) = 3:1:1, 3:2:1, 3:3:1, 3:4:1, 2:2:1 and 4:2:1 were mixed together and stirred at 300 rpm 40 °C for 3.5 h. Then, the synthesized CaAl-Cl LDH were separated by vacuum filtration. In order to remove the soluble impurities, the precipitate was further washed with deionized water and dried at 40 °C for 24 h, finally ground into fine powder. The crystal structure of the precipitates was identified by X-ray diffractometer (XRD, X’Pert PRO MPD, Malvern Panalytical Inc., Malvern, UK) in the range of 10° to 90° using Cu K*α* radiation at 34 kV and 20 mA and the scanning rate of 0.02°/s. The chloride concentration in the filtrate was determined by Inductively Coupled Plasma-mass Spectrometry (XSeries II ICP-MS, Thermo Fisher Scientific Inc., Carlsbad, CA, USA). Chloride ions removal efficiency (R, %) can be obtained from Equation (1):

(1) $$R=\frac{C_{o}-C_{e}}{C_{o}}\times 100\%$$

where *C*_o_ and *C*_e_ represents the initial and final chloride concentration (mg/L) in the solution, respectively.

### 2.2. Chloride Adsorption Kinetics and Isotherms

The CaAl Cl-LDH precursor is synthesized by the reaction interacted by Ca(OH)_2_ and NaAlO_2_ with the optimum Ca/Al ratio, which have the Cl^−^ ion absorbent capability. NaCl solutions (0.12 mol/L, 0.24 mol/L, and 0.48 mol/L) and the CaAl-Cl LDH precursors were mixed together to investigate the chloride adsorption kinetics [10]. At regular intervals (5 min, 10 min, 15 min, 20 min, 25 min, 30 min, 1 h, 2 h, and 3.5 h), 3 mL supernatant was timely filtered and measured by ICP-MS for the analysis of Cl^−^ concentration. The equilibrium chloride adsorption amount *Q*_e_ (mg/g) is determined according to the Equation (2):

(2) $$Q_{e}=\frac{(C_{o}-C_{e})v}{m}$$

where *m* is the mass (g) of the total mass of Ca(OH)_2_ and NaAlO_2_ and *V* is the volume (L) of NaCl solution.

### 2.3. Fixation Stability of Cement Mortar Blended with NaCl Solution

Ordinary Portland cement (PO 42.5) in accordance with Chinese Standard GB175-2007 was used in this study [12]. Three binders were investigated. As shown in Table 1, for binder P1 and P2, NaCl solution (0.12 mol/L) and desalinated water collected from the synthesis of CaAl-Cl LDH were used as the mixing water, respectively. For binder P3, synthesized CaAl precursors were blended in the cement mixture (5% of total cement mass). All samples were cast in molds 4 × 4 × 16 cm^3^. During the first 24 h, all samples were kept in molds inside a climate chamber (RH 95%, 20 °C), then demolded and cured in a saturated lime water for 3 days and 28 days.

Free chloride content was measured by the leaching method described in the Test Code for Hydraulic Concrete (SL352-2006) [13]. Firstly, cement mortar was crushed into small particles (< 5 mm). Secondly, 1.0 g sample together with 100 ± 1 mL deionized water was added into a 250^−^mL beaker. Thirdly, the breaker was fixed onto the oscillators and vibrated for 1 h, 2 h, 3 h, 5 h, 7 h, 10 h, 15 h, 24 h, and 30 h. Fourthly, the solid components were extracted onto the membrane filter with 0.45 μm sieve size via vacuum filtration method. Finally, the chloride content of the filtered extract was analyzed by using ICP measurement at 3 days and 28 days, respectively.

### 2.4. Influence of Inhibitors on the Corrosion Behavior of Steel

Carbon steel (Q235 steel bar, Ø10 mm, and L 90 mm) with the composition of C 0.181%, Mn 0.580%, Si 0.350%, S 0.023%, P 0.012%, and Fe 97.5% was used in this study. Detailed preparation of steel surface is described elsewhere [14]. The steel were ground with SiC paper from 100^#^, 400^#^, 1000^#^, and 1200^#^, then further polished with Al_2_O_3_ polishing powder, finally rinsed with acetone (Aladdin, Shanghai, China). Then, the steel was passivated in the saturated lime water and the ends of the steel connected with copper leads were coated with dense epoxy. The electrolytes are 3.5 wt % NaCl ([Cl^−^] = 0.12 mol/L) saturated Ca(OH)_2_ solution with and without 0.35 wt % CaAl LDH precursor.

The electrochemical measurements were performed by using the electrochemical workstation (Versa STAT 3F, Princeton Applied Research, Oak Ridge, TN, USA) with a typical three-electrode cell. The steel serves as the working electrode, saturated calomel electrode serves as the reference electrode and platinized titanium anode with a working area 1 cm^2^ serving as the counter electrode. The steel was immersed in the electrolytes for 30 min to achieve the state of open circuit potential (OPC) before the measurement. The testing frequency ranged from 10^5^ to 0.1 Hz, and the 10 mV amplitude of the sinusoidal cross-circuit was adopted. All the results of electrochemical testing are the average of three test runs.

## 3. Results and Analysis

### 3.1. Synthesis of CaAl-Cl LDH with Various n(Ca: Al: Cl) Ratio

The formed mechanism of calcium chloroaluminate can be classified into two patterns: A direct chemical reaction among chloride, lime, and calcium aluminate, see Equation (3), and an indirect ion exchange between the OH^−^ ions presented in the interlayers of LDH phase Ca_4_Al_2_ (OH)_14_ and unbounded chloride ions, see Equations (4) and (5) [8].

4Ca^2+^ + 2Al(OH)_4_^−^ + 2Cl^−^ + 4OH^−^ ⇌ Ca_4_Al_2_O_6_Cl_2_·10H_2_O (3)

4Ca^2+^ + 2Al(OH)_4_^−^ + 4OH^-^ ⇌ Ca_4_Al_2_(OH)_14_ (4)

Ca_4_Al_2_(OH)_14_ + 2Cl^−^⇌ Ca_4_Al_2_O_6_Cl_2_·10H_2_O + 2OH^−^ (5)

3Ca_4_Al_2_O_6_Cl_2_·10H_2_O + 2Al(OH)_4_^−^+4OH^−^ ⇌ 4Ca_3_Al_2_(OH)_12_ + 6Cl^−^ + 12H_2_O (6)

3Ca(OH)_2_ + 2Al(OH)_3_ ⇌ Ca_3_Al_2_(OH)_12_ (7)

Ca_3_Al_2_(OH)_12_ + Ca(OH)_2_ + 6H_2_O ⇌ 2Ca_2_Al(OH)_7_·3H_2_O (8)

Since the CaAl LDH precursor is applied as the chloride inhibitor blended in the cementitious materials to prevent the chloride-induced deterioration. Saturated calcium hydroxide is the main ingredient in concrete pore solution and the pH is in range of 13–14 [15]. Many studies have been performed to determine the chloride threshold value leading the corrosion initiation [16,17,18]. The [Cl^−^]/[OH^−^] ratio is defined as the boundary limitation for corrosion occurrence, when the [Cl^−^]/[OH^−^] ratio higher than 0.6, the corrosion occurs [19,20]. Therefore, Ca(OH)_2_ in the pore solution can create an alkaline environment for the interaction of CaAl LDH precursor and chloride ions, and achieve the calcium hydroxides supplementation for dynamic equilibrium. Therefore, we adjusted several Ca:Al:Cl ratio for obtained the optimal Ca:Al:Cl ratio for cementitious materials system. The effect of n (Ca:Al:Cl) ratio on the chloride removal is shown in Table 2. The initial [Cl^−^] concentration was 0.24 mol/L and final Cl concentration was obtained by measuring chloride ions concentration remaining in the filtrate by ICP-MS, then the chloride ions removal efficiency (R, %) can be obtained according to Equation (1). When n(Al/Cl) = 2, the chloride adsorption rate is the most efficient among all n(Ca/Cl) ratios. Additionally, with n(Al/Cl) > 2, the chloride adsorption rate is generally decreased, which is mainly due to the precipitates formed in the first step could further react with the excessive NaAlO_2_ and then more soluble aluminum-chloride-hydroxide complexes will be formed in the solution [21]. Correspondingly, with n(Al/Cl) = 2, n(Ca/Cl) < 2, there is an obvious decrease in chloride adsorption rate, which is mainly due to the lack of calcium to react with the aluminum in the solution to form LDH phase Ca_4_Al_2_ (OH)_14_. Generally, if the formation of LDH phase Ca_4_Al_2_ (OH)_14_ is the only critical issue that affects the chloride adsorption, n(Al/Cl) ratio should equal 2.0 and n(Ca/Cl) should equal 3.0. This deviation of the observed efficiency from that expected for absorption of chloride should be caused by the formation of the other solid phase which will be discussed in detail below. Therefore, these results show that, in the case of chloride form of hydrocalumite, n(Ca:Al:Cl) = 3:2:1 is considered to be the optimal proportional and the chloride ions reduction was 87.06%.

**Figure 1:**
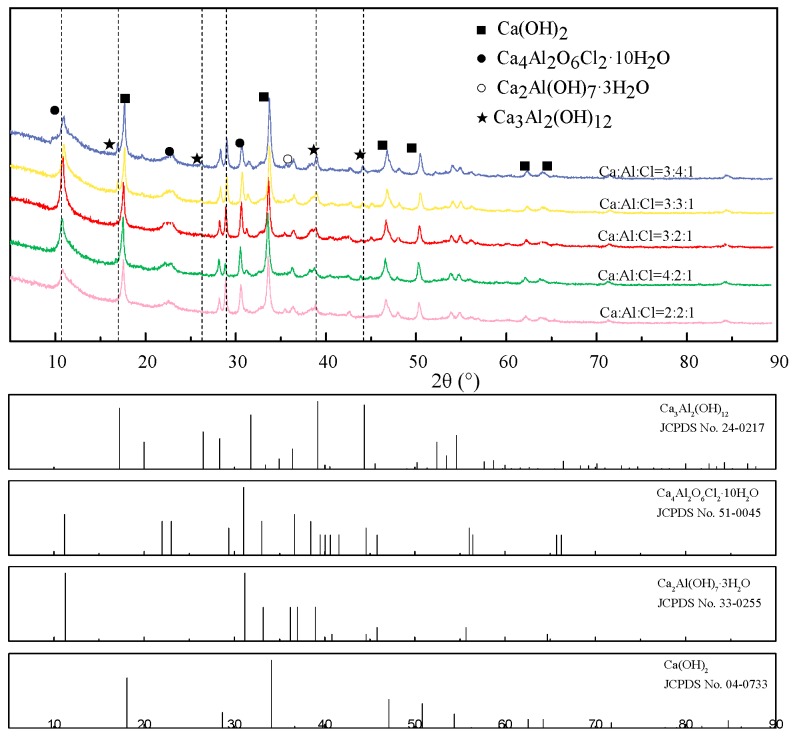
XRD patterns of precipitations after adsorption of chloride vs. various n (Ca:Al:Cl) ratios.

To clarify the hypothesis discussed above, the chemical compositions of precipitations are identified by XRD analysis, see Figure 1. As shown in Figure 1, diffraction peaks of synthesized CaAl-Cl LDH with various Ca/Al ratio are in good agreement with Ca_3_Al_2_(OH)_12_ (JCPDS NO. 24-0217), Ca_4_Al_2_O_6_Cl_2_·10H_2_O (JCPDS NO. 51-0045), Ca_2_Al(OH)_7_·3H_2_O (JCPDS NO. 33-0255), and Ca(OH)_2_ (JCPDS NO. 04-0733). The diffraction peaks at 11.67° corresponding to Ca_4_Al_2_O_6_Cl_2_·10H_2_O is weakened with the excessive amount of NaAlO_2_. This is mainly due to the transformation of Ca_4_Al_2_O_6_Cl_2_·10H_2_O into Ca_3_Al_2_(OH)_12_, see Equation (6). In consequence, the chloride ions can be chemically released by the reaction with soluble Al(OH)_4_^−^. As for the excessive amount of Ca(OH)_2_, the diffraction peaks at 44.5°, corresponding to Ca_3_Al_2_(OH)_12_, is weakened, which is due to the transformation of Ca_3_Al_2_(OH)_12_ into Ca_2_Al(OH)_7_·3H_2_O, see Equations (7) and (8). According to the crystallinity calculation results by using JADE software, the calculated crystallinity of CaAl-Cl LDH with various Ca: Al: Cl ratios was determined and can be regarded as the semi-quantitative method for considering the optimal proportional for the formation of CaAl-Cl LDH. According to the above discussion, n(Ca): n(Al): n(Cl) = 3:2:1 is considered to be the optimal proportion for the formation of CaAl-Cl LDH.

### 3.2. Kinetic Study and Adsorption Isotherm

**Figure 2:**
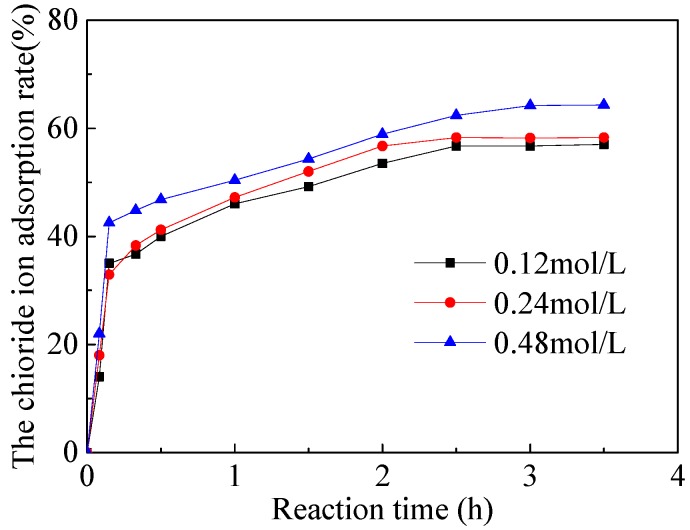
Effect of contact time on the adsorption of Cl^−^ onto the CaAl-Cl LDH precursors with [Cl^−^] = 0.12 mol/L, 0.24 mol/L and 0.48 mol/L.

The kinetics of Cl^−^ adsorption by CaAl-Cl LDH precursors is shown in Figure 2. The results reveal that CaAl-Cl LDH precursors exhibits a higher adsorption rate (32–43% chloride adsorbed) at the first 10 min. Then, the adsorption rates slow down and tend to plat after 10 min. Due to the chloride adsorption by CaAl-Cl LDH precursors belongs to the underlying mechanism of chemisorption. Therefore, the pseudo-second-order kinetic model depicted in Equation (9) is applied to characterize the adsorption process of the chloride adsorption process:

(9) $$\frac{dQ_{t}}{dt}=k_{2}{\left(Q_{e}-Q_{t}\right)}^{2}$$

where *Q*_e_ (mg/g) is the chloride adsorbed capacity on the CaAl-Cl LDH precursors at equilibrium, *Q*_t_ (mg/g) is the chloride adsorbed amount at time t, *k_2_* (g/mg min^−1^) is the second-order adsorption constant.

Equation (9) can be reduced to a simple formula as:

(10) $$\frac{t}{Q_{t}}=\frac{1}{k_{2}Q_{e}^{2}}+\frac{t}{Q_{e}}$$

The kinetic parameters and correlation coefficient (*R*^2^) are summarized in Table 3. The calculated adsorption capacities (*Cal-Q*_e_) by using pseudo-second-order model are very close to the experimental values (*Exp-Q*_e_). The results indicate that pseudo-second-order model can be applied to describe the adsorption process of the chloride on CaAl-Cl LDH precursors.

**Figure 3:**
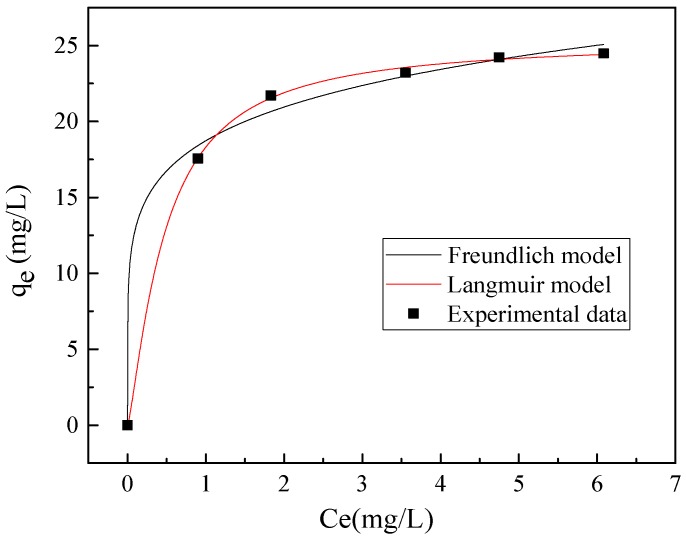
Isotherm for adsorption of chloride on CaAl-Cl LDH precursors.

Langmuir and Freundlich isotherm models are widely applied in the absorption process [22]. In this study, both models are comparatively adopted to analyze and explain the chloride absorption process, see Figure 3. Langmuir adsorption isotherm model is more fit for determining the adsorption rate and capacity of the monolayer chloride ions on the outer surface of the adsorbent, but failed to determine, if any, the further or other types of adsorption. The equilibrium distribution of ions from the solid to the liquid phases can be represented by the Langmuir model [23]:

(11) $$Q_{e}=\frac{k_{L}Q_{m}C_{e}}{1+k_{L}C_{e}}$$

where *C*_e_ is the equilibrium mass concentration of the CaAl-Cl LDH precursors (mg/L), *Q*_m_ is the maximum adsorption amount of the chloride ion by CaAl-Cl LDH precursors (mg/g), and *k*_L_ is Langmuir isotherm constant (L/mg).

Other than Langmuir model, Freundlich adsorption isotherm model Equation (12) is commonly used to describe the adsorption characteristic among the heterogeneous surfaces [24]:

(12) $$Q_{e}=k_{F}C_{e}^{1/n}$$

where *k_F_* is Freundlich isotherm constant (mg/g), *n* is the adsorption intensity. *k_F_* and n could be determined by data fitting. The correlation coefficients *R*^2^ of the Freundlich and Langmuir model are 0.907 and 0.989, respectively. Langmuir adsorption isotherm is known as the formation process of a monolayer metal ions on the outer surface of the adsorbent, and Freundlich adsorption isotherm is usually applied to describe the adsorption characteristics of the heterogeneous surface [25]. Therefore, the chloride ions were adsorbed first on the LDH precursors edges, surface region, and then the interlayer space via diffusion [26,27]. Therefore, the Langmuir model is more suitable to describe the adsorption process of chloride ions on the CaAl LDH precursors.

**Figure 4:**
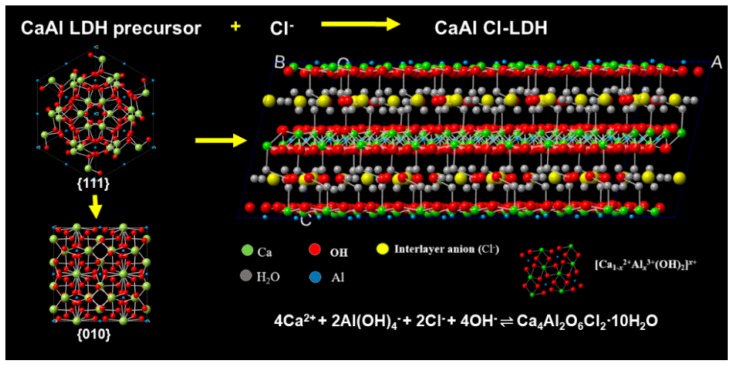
Calculated structures of CaAl LDH precursor and CaAl-Cl LDH. Color key: Green, blue and yellow spheres represent Ca, Al and Cl atoms, respectively. Red and grey spheres represent H_2_O and OH group.

Figure 4 presents the calculated structures of CaAl LDH precursor and CaAl-Cl LDH visualized by Material studio. The cubic structure of CaAl LDH precursor was presented as the 24-side deltoidal icositetrahedron from {010} faces [28]. Furthermore, hydroxyl groups is served as a part of Al(OH)_4_^−^ groups in the bulk structures. However, with the adsorption of chloride ions by CaAl LDH precursor, it can be noticed that the atomic structure of CaAl-Cl LDH was shown as the layered crystals with octahedral structure as the skeleton and chloride ions as the interlayered anion from {110} faces. The transformation of defined morphology to newly formed layered polyhedral structure is ascribed to as the molecular recognition between the crystal surfaces and the adsorption anions [28]. Taking the above analysis into account, the adsorption mechanism during the chloride ions adsorption process can be ascribed to the co-precipitation interacted between Cl^−^ and the hydrolysis product of CaAl LDH precursors.

### 3.3. Chloride Leaching Test

**Figure 5:**
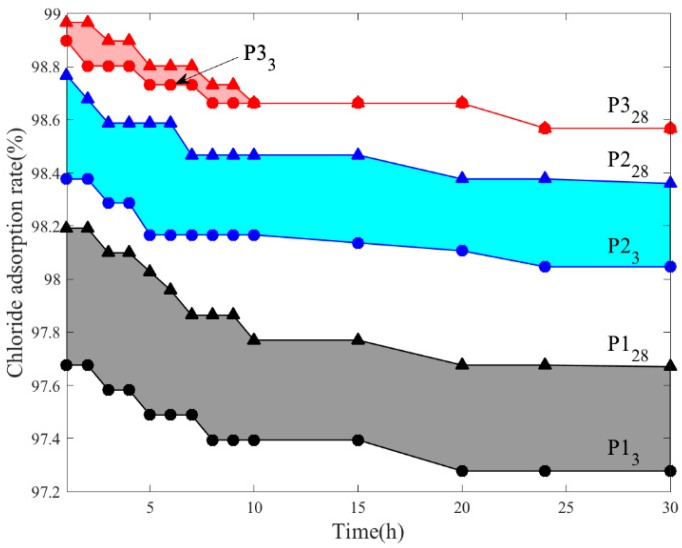
The chloride adsorption ratio of P1, P2, and P3 at 3d and 28d.

In order to testify the stability of chloride ions in the mortar, chloride ions leaching test was performed according to Test Code for Hydraulic Concrete (SL352-2006)**.** Figure 5 shows the chloride immobilization rate in mortar P1, P2, and P3 at three days and 28 days, respectively. The results indicate that the chloride ions adsorption rate of mortar blended with CaAl-Cl LDH precursors is the highest. This is mainly due to the chloride ions could be captured by CaAl-Cl LDH precursors and then turn into CaAl-Cl LDH. Small gaps are presented between P3 at three days and 28 days, and 98.3% chloride ions have been immobilized in cementitious materials, which indicates chloride ions have been efficiently adsorbed by pre-blended CaAl-Cl LDH precursors in the early age and tiny parts of them will be further absorbed by the gradually formed cement hydration products. However, these further absorbed parts (Red region) will be easily released during the first 10 h leaching test period. The chloride ion leaching content of mortar P2 or P3 at 3d was higher than that of the same mortar at 28d. Correspondingly, the marked reduction of chloride ions adsorption rate P2 (Blue region) and P1 (Black region) indicates that chloride ions could be immobilized by the hydration products in the cement matrix [29]. The chloride binding mechanisms of the hydrated cement mortar have been summarized into two explanations. The chloride ions in the pore solution can easily enter into the interlayer space among the C–S–H gels and partly replace with OH^−^ ions to maintain the charge balance [8,30,31,32,33]. Additionally, chemical reaction between the chloride ions and the AFm phases is another mechanism for chloride immobilization [34]. In particular, the AFm phase is a typical calcium derivative of double hydroxides (LDHs) family, which can adsorb chloride ions by forming CaAl-Cl LDH. Therefore, it can be concluded that the mortar blended with CaAl-Cl LDH precursors could effectively prevent chloride ions ingress. This study provides additional theoretical and technical foundations for the feasibility of seawater mixing concrete.

### 3.4. Electrochemical Evaluation

**Figure 6:**
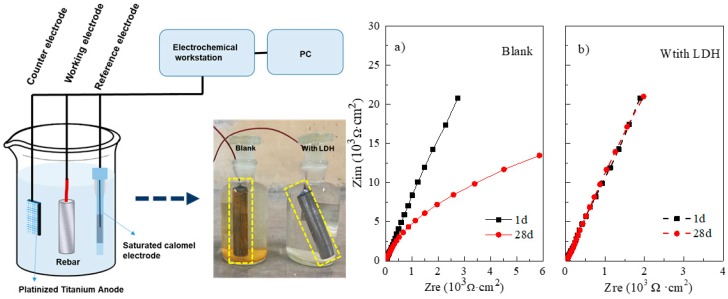
The schematic of the electrochemical (left); the corrosion state of steel bars immersed in two electrolytes after 28d (middle); the impedance spectra plots of steel immersed in two electrolytes (with/without inhibitor) at 1d and 28d (**a**,**b**).

In the Figure 6, the surface of the sample immersed in reference system (3.5% NaCl saturated Ca(OH)_2_ solution without inhibitor) for 28d is fully covered by ions rust. Correspondingly, the surface of the sample immersed in the system with inhibitor is still brightness. Therefore, electrochemical measurement by EIS test was performed to further evaluate the corrosion state of the steel. Figure 6a) is the impedance spectra plots of steel immersed in the reference system at 1d and 28d. The initial impedance at 24 h is quite large. This is due to the passive film was formed on the surface of the steel in the saturated lime water [18]. However, there is a relative decrease of the diameter of semi-circle in the Nyquist plots at 28 days, which reveals the occurrence of corrosion activities on the surface of the steel [19]. This is mainly due to the protective film destroyed by forming soluble FeCl_2_. Correspondingly, Figure 6b is the impedance spectra plots of steel immersed in the system with inhibitor at 1d and 28d. And these two impedance spectra curves show identical electrochemical behaviors. This is due to the chloride ions adsorbed by CaAl-Cl LDH precursor by forming CaAl-Cl LDH and consequently prevent the further corrosion. Therefore, it can be concluded that CaAl-Cl LDH precursor can be served as an efficiently inhibitor hinders the corrosion actively.

## 4. Conclusions

The following conclusions can be drawn based on the present laboratory investigation.

1. Chloride ions could be effectively adsorbed by CaAl LDH precursor. The optimal chloride ion removal rate was 87.06% due to the formation of hydrocalumite with Ca: Al: Cl = 3: 2: 1.
2. The adsorption process could be well described by the pseudo-second-order model and Langmuir model. 98.3% chloride ions can be rapidly captured in cement mortar blended with CaAl-Cl LDH precursor and cannot be easily released again.
3. The inhibition performance of steel in the electrolytes with/without CaAl LDH precursor was investigated by using electrochemical measurements. The results indicates that CaAl LDH precursor protect the passive film on steel surface by chloride adsorption.
4. This research indicates that CaAl-Cl LDH precursor is a potential and rapid adsorbent for immobilize chloride from sodium chloride water that prevents chloride-induced deterioration in reinforced concrete or mortar.

## Figures and Tables

**Table 1:** Mix proportions of cement mortar (g).

Binder	OPC	Sand	Absorb-Mixture	NaCl Solution	Desalinated Water
P1	450	1350	/	225	/
P2	450	1350	/	/	225
P3	427.5	1350	22.5	225	/

**Table 2:** Ion concentration of filtrates by ICP-MS analysis.

n(Ca:Al:Cl)	pH	n(Cl^−^) g/L	n(Al^3+^) g/L	n(Ca^2+^) g/L	R %
3:1:1	12.65	1.707	0.400	0.053	79.96%
3:2:1	12.70	1.102	2.836	0.039	87.06%
3:3:1	12.67	1.511	5.839	0.049	82.27%
3:4:1	12.78	1.816	9.185	0.037	78.69%
2:2:1	12.66	1.807	3.579	0.032	78.79%
4:2:1	12.72	1.141	2.060	0.025	86.61%

**Table 3:** Comparison between the measured and estimated *Q*_e_ from the pseudo-second-order model.

	*Exp-Q*_e_ (mg/g)	*k*_2_ (g/mg·min)	*Cal-Q*_e_ (mg/g)	*R* ^2^
0.12 mol/L	22.7	0.0030	23.1	0.9997
0.24 mol/L	22.2	0.0029	23.8	0.9979
0.48 mol/L	25.5	0.0024	25.7	0.9998
